# PP48 Analysis Of CONITEC’s Recommendations According The Triple Burden Of Diseases Groups From 2012 To 2023

**DOI:** 10.1017/S0266462325102675

**Published:** 2025-12-29

**Authors:** Clementina Corah Lucas Prado, Luciene Bonan, Priscila Louly, Mariana Caramaschi, Yara Marques

## Abstract

**Introduction:**

Brazil faces a triple burden of disease—infectious diseases, chronic non-communicable diseases, and external causes—that demand effective public health policies and access to the healthcare system. Analyzing the health technology assessment process allows us to determine whether the health needs of the Brazilian population are being met by new technologies or whether they contribute to increasing health inequalities.

**Methods:**

This descriptive, exploratory study was based on an evaluation of the National Committee for Health Technology Incorporation (CONITEC) recommendations on technologies from 2012 to 2023, carried out year by year using the burden of diseases reference. The aim was to evaluate and classify each recommendation according to the triple burden of disease groups, such as infectious diseases (group one), chronic non-communicable diseases (group two), and external causes (group three), plus one more (group four) that was adapted for this study from group one to emphasize neglected tropical diseases (NTD).

**Results:**

Each of CONITEC’s 822 recommendations was evaluated and classified into one of the groups: 151 technologies for group one, especially HIV/AIDS, hepatitis, tuberculosis, COVID-19 and life cycles (18% of total); 645 technologies for group two, especially in the medical specialties of oncology, rheumatology, neurology, pulmonology, medical genetics, and cardiology (78% of total) and 41 percent of which were for rare diseases; 10 technologies for group three; and 12 technologies for group four, covering only four diseases (leprosy, leishmaniasis, Chagas disease, and dengue fever).

**Conclusions:**

Although 18 percent of CONITEC’s recommendations included priority infectious diseases in Brazil, less than two percent related to NTD. For chronic diseases, 41 percent were for rare diseases, without treatment for a long time, but with a high budgetary impact that affects the financial sustainability of the SUS. This may reflect the interests of manufacturers rather than health policies.

